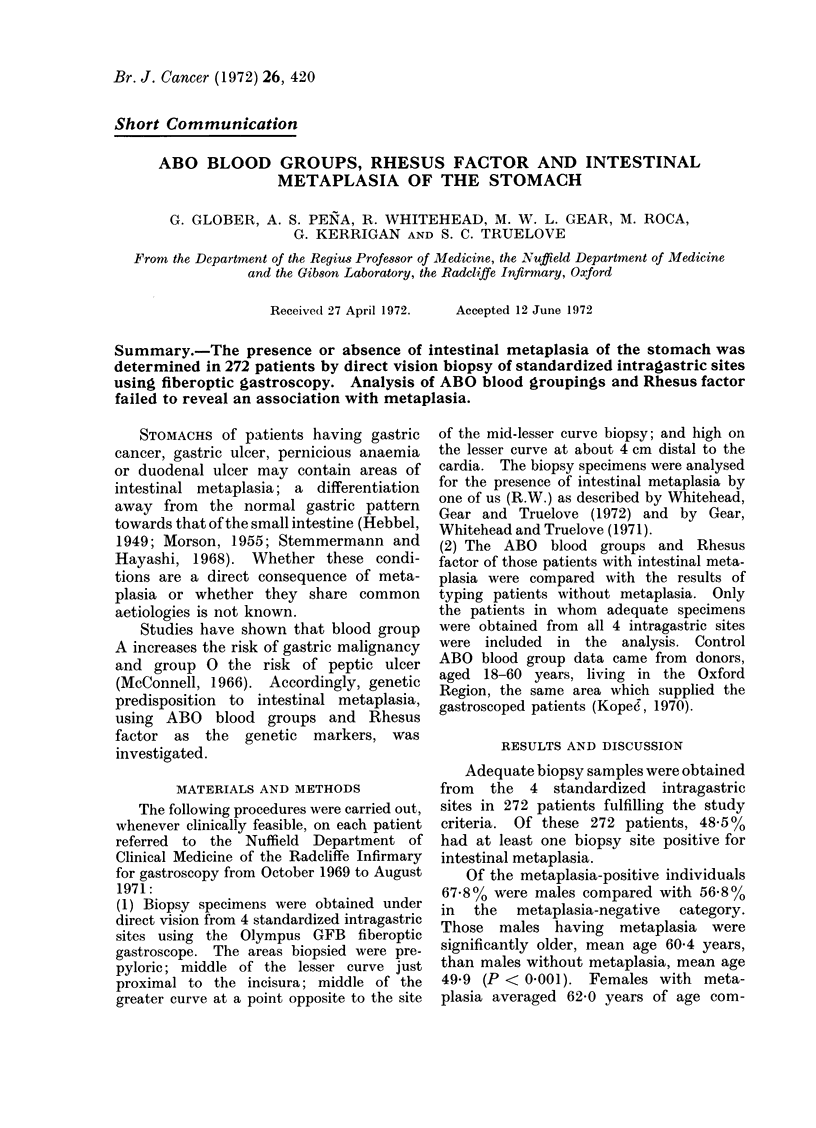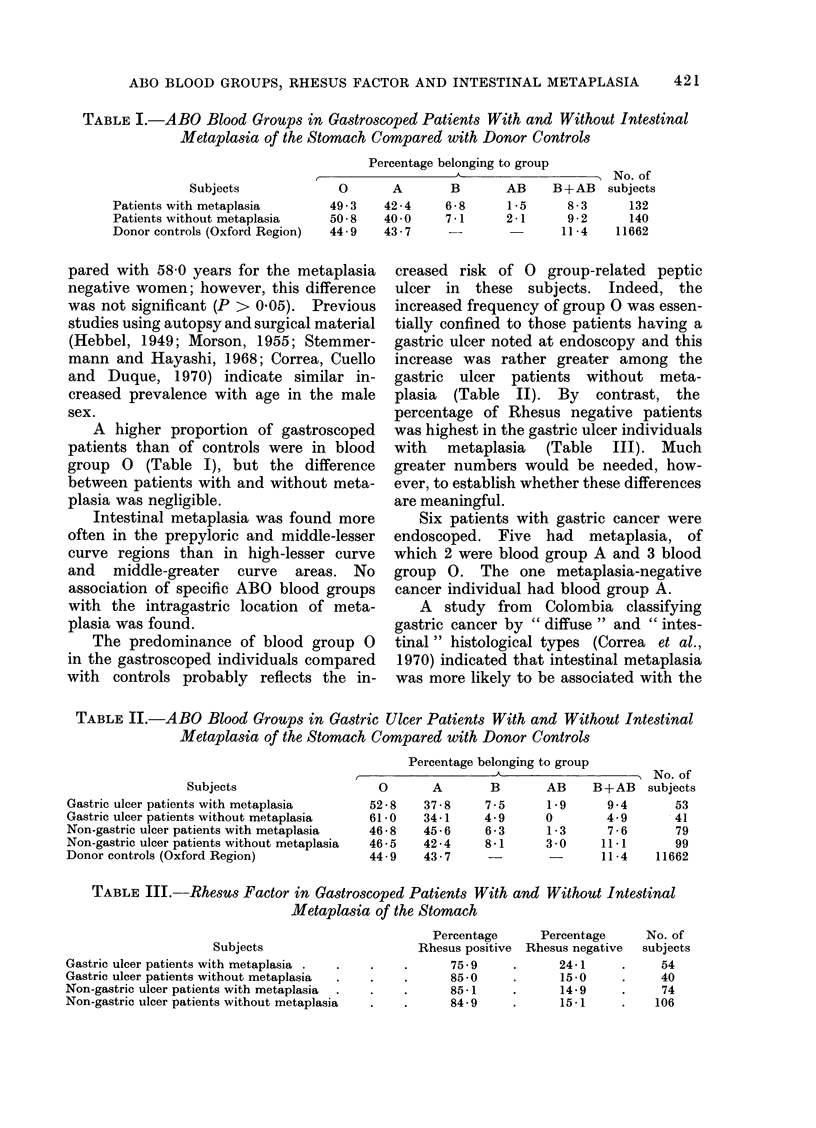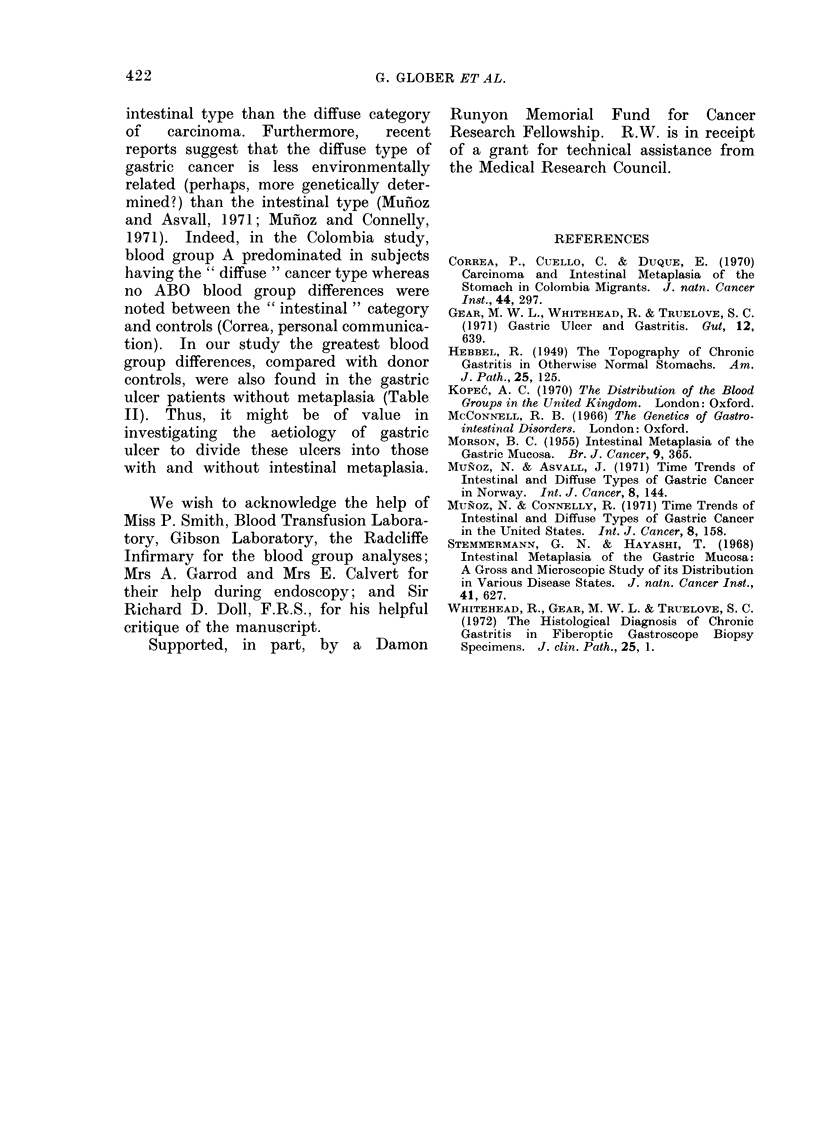# ABO Blood Groups, Rhesus Factor and Intestinal Metaplasia of the Stomach

**DOI:** 10.1038/bjc.1972.55

**Published:** 1972-10

**Authors:** G. Glober, A. S. Peña, R. Whitehead, M. W. L. Gear, M. Roca, G. Kerrigan, S. C. Truelove

## Abstract

The presence or absence of intestinal metaplasia of the stomach was determined in 272 patients by direct vision biopsy of standardized intragastric sites using fiberoptic gastroscopy. Analysis of ABO blood groupings and Rhesus factor failed to reveal an association with metaplasia.


*Br. J. Cancer* (1972) **26**, 420

***Short Communication***

### ABO BLOOD GROUPS, RHESUS FACTOR AND INTESTINAL METAPLASIA OF THE STOMACH

G. GLOBER, A. S. PEÑA, R. WHITEHEAD, M. W. L. GEAR, M. ROCA, G. KERRIGAN AND S. C. TRUELOVE

*From the Department of the Regius Professor of Medicine, the Nuffield Department of Medicine and the Gibson Laboratory, the Radcliffe Infirmary, Oxford*

Received 27 April 1972. Accepted 12 June 1972

**Summary.—The presence or absence of intestinal metaplasia of the stomach was determined in 272 patients by direct vision biopsy of standardized intragastric sites using fiberoptic gastroscopy. Analysis of ABO blood groupings and Rhesus factor failed to reveal an association with metaplasia.**

STOMACHS of patients having gastric cancer, gastric ulcer, pernicious anaemia or duodenal ulcer may contain areas of intestinal metaplasia; a differentiation away from the normal gastric pattern towards that of the small intestine (Hebbel, 1949; Morson, 1955; Stemmermann and Hayashi, 1968). Whether these conditions are a direct consequence of metaplasia or whether they share common aetiologies is not known.

Studies have shown that blood group A increases the risk of gastric malignancy and group O the risk of peptic ulcer (McConnell, 1966). Accordingly, genetic predisposition to intestinal metaplasia, using ABO blood groups and Rhesus factor as the genetic markers, was investigated.

### MATERIALS AND METHODS

The following procedures were carried out, whenever clinically feasible, on each patient referred to the Nuffield Department of Clinical Medicine of the Radcliffe Infirmary for gastroscopy from October 1969 to August 1971:

(1) Biopsy specimens were obtained under direct vision from 4 standardized intragastric sites using the Olympus GFB fiberoptic gastroscope. The areas biopsied were prepyloric; middle of the lesser curve just proximal to the incisura; middle of the greater curve at a point opposite to the site of the mid-lesser curve biopsy; and high on the lesser curve at about 4 cm distal to the cardia. The biopsy specimens were analysed for the presence of intestinal metaplasia by one of us (R.W.) as described by Whitehead, Gear and Truelove (1972) and by Gear, Whitehead and Truelove (1971).

(2) The ABO blood groups and Rhesus factor of those patients with intestinal metaplasia were compared with the results of typing patients without metaplasia. Only the patients in whom adequate specimens were obtained from all 4 intragastric sites were included in the analysis. Control ABO blood group data came from donors, aged 18–60 years, living in the Oxford Region, the same area which supplied the gastroscoped patients (Kopeč, 1970).

### RESULTS AND DISCUSSION

Adequate biopsy samples were obtained from the 4 standardized intragastric sites in 272 patients fulfilling the study criteria. Of these 272 patients, 48·5% had at least one biopsy site positive for intestinal metaplasia.

Of the metaplasia-positive individuals 67·8% were males compared with 56·8% in the metaplasia-negative category. Those males having metaplasia were significantly older, mean age 60·4 years, than males without metaplasia, mean age 49·9 ($P < 0{\cdot}001$). Females with metaplasia averaged 62·0 years of age com-

TABLE I.—*ABO Blood Groups in Gastroscoped Patients With and Without Intestinal Metaplasia of the Stomach Compared with Donor Controls*

| Subjects | Percentage belonging to group O | A | B | AB | B+AB | No. of subjects |
|---|---|---|---|---|---|---|
| Patients with metaplasia | 49·3 | 42·4 | 6·8 | 1·5 | 8·3 | 132 |
| Patients without metaplasia | 50·8 | 40·0 | 7·1 | 2·1 | 9·2 | 140 |
| Donor controls (Oxford Region) | 44·9 | 43·7 | — | — | 11·4 | 11662 |

pared with 58·0 years for the metaplasia negative women; however, this difference was not significant ($P > 0{\cdot}05$). Previous studies using autopsy and surgical material (Hebbel, 1949; Morson, 1955; Stemmermann and Hayashi, 1968; Correa, Cuello and Duque, 1970) indicate similar increased prevalence with age in the male sex.

A higher proportion of gastroscoped patients than of controls were in blood group O (Table I), but the difference between patients with and without metaplasia was negligible.

Intestinal metaplasia was found more often in the prepyloric and middle-lesser curve regions than in high-lesser curve and middle-greater curve areas. No association of specific ABO blood groups with the intragastric location of metaplasia was found.

The predominance of blood group O in the gastroscoped individuals compared with controls probably reflects the increased risk of O group-related peptic ulcer in these subjects. Indeed, the increased frequency of group O was essentially confined to those patients having a gastric ulcer noted at endoscopy and this increase was rather greater among the gastric ulcer patients without metaplasia (Table II). By contrast, the percentage of Rhesus negative patients was highest in the gastric ulcer individuals with metaplasia (Table III). Much greater numbers would be needed, however, to establish whether these differences are meaningful.

Six patients with gastric cancer were endoscoped. Five had metaplasia, of which 2 were blood group A and 3 blood group O. The one metaplasia-negative cancer individual had blood group A.

A study from Colombia classifying gastric cancer by " diffuse " and " intestinal " histological types (Correa *et al.*, 1970) indicated that intestinal metaplasia was more likely to be associated with the

TABLE II.—*ABO Blood Groups in Gastric Ulcer Patients With and Without Intestinal Metaplasia of the Stomach Compared with Donor Controls*

| Subjects | Percentage belonging to group O | A | B | AB | B+AB | No. of subjects |
|---|---|---|---|---|---|---|
| Gastric ulcer patients with metaplasia | 52·8 | 37·8 | 7·5 | 1·9 | 9·4 | 53 |
| Gastric ulcer patients without metaplasia | 61·0 | 34·1 | 4·9 | 0 | 4·9 | 41 |
| Non-gastric ulcer patients with metaplasia | 46·8 | 45·6 | 6·3 | 1·3 | 7·6 | 79 |
| Non-gastric ulcer patients without metaplasia | 46·5 | 42·4 | 8·1 | 3·0 | 11·1 | 99 |
| Donor controls (Oxford Region) | 44·9 | 43·7 | — | — | 11·4 | 11662 |

TABLE III.—*Rhesus Factor in Gastroscoped Patients With and Without Intestinal Metaplasia of the Stomach*

| Subjects | Percentage Rhesus positive | Percentage Rhesus negative | No. of subjects |
|---|---|---|---|
| Gastric ulcer patients with metaplasia | 75·9 | 24·1 | 54 |
| Gastric ulcer patients without metaplasia | 85·0 | 15·0 | 40 |
| Non-gastric ulcer patients with metaplasia | 85·1 | 14·9 | 74 |
| Non-gastric ulcer patients without metaplasia | 84·9 | 15·1 | 106 |

intestinal type than the diffuse category of carcinoma. Furthermore, recent reports suggest that the diffuse type of gastric cancer is less environmentally related (perhaps, more genetically determined?) than the intestinal type (Muñoz and Asvall, 1971; Muñoz and Connelly, 1971). Indeed, in the Colombia study, blood group A predominated in subjects having the " diffuse " cancer type whereas no ABO blood group differences were noted between the " intestinal " category and controls (Correa, personal communication). In our study the greatest blood group differences, compared with donor controls, were also found in the gastric ulcer patients without metaplasia (Table II). Thus, it might be of value in investigating the aetiology of gastric ulcer to divide these ulcers into those with and without intestinal metaplasia.

We wish to acknowledge the help of Miss P. Smith, Blood Transfusion Laboratory, Gibson Laboratory, the Radcliffe Infirmary for the blood group analyses; Mrs A. Garrod and Mrs E. Calvert for their help during endoscopy; and Sir Richard D. Doll, F.R.S., for his helpful critique of the manuscript.

Supported, in part, by a Damon Runyon Memorial Fund for Cancer Research Fellowship. R.W. is in receipt of a grant for technical assistance from the Medical Research Council.

### REFERENCES

CORREA, P., CUELLO, C. & DUQUE, E. (1970) Carcinoma and Intestinal Metaplasia of the Stomach in Colombia Migrants. *J. natn. Cancer Inst.*, **44**, 297.

GEAR, M. W. L., WHITEHEAD, R. & TRUELOVE, S. C. (1971) Gastric Ulcer and Gastritis. *Gut*, **12**, 639.

HEBBEL, R. (1949) The Topography of Chronic Gastritis in Otherwise Normal Stomachs. *Am. J. Path.*, **25**, 125.

KOPEĆ, A. C. (1970) *The Distribution of the Blood Groups in the United Kingdom.* London: Oxford.

McCONNELL, R. B. (1966) *The Genetics of Gastro-intestinal Disorders.* London: Oxford.

MORSON, B. C. (1955) Intestinal Metaplasia of the Gastric Mucosa. *Br. J. Cancer*, **9**, 365.

MUÑOZ, N. & ASVALL, J. (1971) Time Trends of Intestinal and Diffuse Types of Gastric Cancer in Norway. *Int. J. Cancer*, **8**, 144.

MUÑOZ, N. & CONNELLY, R. (1971) Time Trends of Intestinal and Diffuse Types of Gastric Cancer in the United States. *Int. J. Cancer*, **8**, 158.

STEMMERMANN, G. N. & HAYASHI, T. (1968) Intestinal Metaplasia of the Gastric Mucosa: A Gross and Microscopic Study of its Distribution in Various Disease States. *J. natn. Cancer Inst.*, **41**, 627.

WHITEHEAD, R., GEAR, M. W. L. & TRUELOVE, S. C. (1972) The Histological Diagnosis of Chronic Gastritis in Fiberoptic Gastroscope Biopsy Specimens. *J. clin. Path.*, **25**, 1.